# Nondestructive Inspection and Quantification of Select Interface Defects in Honeycomb Sandwich Panels

**DOI:** 10.3390/ma17112772

**Published:** 2024-06-06

**Authors:** Mahsa Khademi, Daniel P. Pulipati, David A. Jack

**Affiliations:** Department of Mechanical Engineering, Baylor University, Waco, TX 76798, USA; mahsa_khademi1@baylor.edu (M.K.); daniel_pulipati@alumni.baylor.edu (D.P.P.)

**Keywords:** nondestructive testing, honeycomb core composite, defect identification, defect quantification, carbon fiber composite

## Abstract

Honeycomb sandwich panels are utilized in many industrial applications due to their high bending resistance relative to their weight. Defects between the core and the facesheet compromise their integrity and efficiency due to the inability to transfer loads. The material system studied in the present paper is a unidirectional carbon fiber composite facesheet with a honeycomb core with a variety of defects at the interface between the two material systems. Current nondestructive techniques focus on defect detectability, whereas the presented method uses high-frequency ultrasound testing (UT) to detect and quantify the defect geometry and defect type. Testing is performed using two approaches, a laboratory scale immersion tank and a novel portable UT system, both of which utilize only single-side access to the part. Coupons are presented with defects spanning from 5 to 40 mm in diameter, whereas defects in the range of 15–25 mm and smaller are considered below the detectability limits of existing inspection methods. Defect types studied include missing adhesive, unintentional foreign objects that occur during the manufacturing process, damaged core, and removed core sections. An algorithm is presented to quantify the defect perimeter. The provided results demonstrate successful defect detection, with an average defect diameter error of 0.6 mm across all coupons studied in the immersion system and 1.1 mm for the portable system. The best accuracy comes from the missing adhesive coupons, with an average error of 0.3 mm. Conversely, the worst results come from the missing or damaged honeycomb coupons, with an error average of 0.7 mm, well below the standard detectability levels of 15–25 mm.

## 1. Introduction

Honeycomb sandwich panels represent an integral component of structures in various industries, including aerospace, automotive, naval, sporting, and construction (see, e.g., [1,2]), owing to a variety of factors, such as their exceptional strength-to-weight ratio, insulation capabilities, and structural versatility. Comprising two thin, high-strength face sheets bonded to a lightweight core material, such as aluminum or Nomex honeycomb, these panels offer significant advantages. However, their susceptibility to defects during manufacturing or in-service poses challenges to their structural integrity and operational efficiency. One example of note was the failure of the X-33 sandwich composite liquid hydrogen tank [3]. Defects in honeycomb sandwich panels arise from various sources, including material imperfections, manufacturing process variability, environmental factors, and mechanical damage (see, e.g., [4,5]). These defects may manifest as voids, delaminations, disbonds, cracks, water ingress, or core crushes, often degrading the mechanical properties and durability of the panels (see, e.g., [6,7,8,9]). Detecting and characterizing these defects are essential for ensuring the safety, reliability, and cost-effectiveness of structures incorporating honeycomb sandwich panels.

Multiple nondestructive testing techniques exist for inspecting defects in honeycomb structures, such as eddy current testing (ECT) [10,11,12,13,14], ultrasonic testing (UT), phased array inspection (PA) [15], thermography testing [9,16,17,18,19,20], acoustic emission testing Shearography [21], and X-ray computed tomography (XCT) [22,23,24,25]. However, these methods often have limitations in terms of sensitivity, accuracy, and efficiency, particularly when dealing with hidden or subsurface defects, complex geometries, and different defect materials. As a result, there is a growing demand for advanced nondestructive evaluation (NDE) techniques capable of detecting and characterizing defects in honeycomb sandwich panels with higher sensitivity and resolution.

Many interesting studies have been conducted on debonding and defect detection in honeycomb sandwich composites using the eddy current testing method. Hagemaier [10] used eddy current conductivity testing to detect core defects in an aluminum-brazed titanium honeycomb structure. He et al. [11] employed eddy current testing to identify defects intentionally embedded between the facesheet and core, simulating delamination. However, their detection was only qualitative rather than quantitative. The study by Underhill et al. [12] investigated the use of eddy current arrays for detecting damage, including disbonding and core defects in sandwich panels with carbon fiber-reinforced polymer (CFRP) facesheets. While they successfully detected core crush or indentation in the honeycomb core, they were unable to quantify these defects. Notably, the effectiveness of defect detection relies on the probe size; where larger probes often result in blurry C-scans of defects. T. Rellinger et al. [13] presented a method combining eddy current, thermography, and 3D laser scanning for inspecting sandwich panel low-velocity impacts to simulate different types of defects. They were only able to detect and measure crushed core defects using eddy current testing without success in identifying other types of defects, such as disbonds and delaminations. Recently, Ren et al. [14] utilized eddy current testing on an aluminum honeycomb sandwich structure with CFRP panels to detect and identify core defects, including wall fractures and core wrinkles. Impact damage detection was also conducted, revealing that as impact energy increased, the affected area in the C-scan image became more blurred and the minimal signal decreased, although the area of damage did not always correspondingly increase. The eddy current method’s limited penetration depth restricts its effectiveness for detecting defects deep within materials. Additionally, it is primarily suitable for conductive materials, posing challenges for inspecting non-conductive or low-conductivity materials [26].

There are also studies focused on finding defects in honeycomb sandwich panels by thermography. Qin and Bao [16] used thermography to detect defects in a honeycomb sandwich panel with an aluminum core. Their detection focused on identifying defects in the honeycomb cells near the panel’s edges. Ibarra-Castanedo et al. [17] utilized three different types of thermography to investigate honeycomb sandwich structure. They concluded that pulse thermography yielded the fastest results but yielded inconsistent results due to nonuniform heating and environmental reflections. Usamentiaga et al. [18] studied holes in a reinforced honeycomb sandwich panel using active thermography. They detected the holes’ presence and were able to estimate the distance from the holes to the reinforced region. Zhao et al. [19] utilized lock-in thermography for disbond detection in titanium alloy honeycomb sandwich panels. They simulated a disbond using a cylindrical air gap with an 18 mm diameter. Hu et al. [20] detected and classified different types of defects in honeycomb sandwich panels, specifically 15 mm and 30 mm diameter flaws. Their model sensitivity was around 90% for water and oil ingress and around 70% for debonding and adhesive pooling defects. The effectiveness of defect detection in thermography is compromised by issues like uneven heating and coil shielding. Moreover, the rapid decay of the temperature signals with increased detection depth limits the range and quantitative accuracy for internal defects in structures, presenting challenges in detecting micro-defects critical for health monitoring [27].

Shearography nondestructive testing (NDT) is finding considerable interest in the detection of flaws within honeycomb sandwich panels. For example, Guo et al. [21] showed that using Shearography, they could detect circular and rectangular defects, with the size of detectability being strongly correlated to the depth at which the defect occurs. In addition, Lobanov et al. [28] showed that Shearography with a combined vacuum load is a reliable method to detect different sizes of defects in titanium honeycomb panels. Revel et al. [29] employed a Wavelet Transform algorithm to quantitatively estimate the delamination size through Shearography inspection. They tested a damaged sandwich panel consisting of a 24 mm honeycomb core and a 1.5 mm thick fiberglass skin. Their coupon featured a set of circular defects with a nominal diameter of 24 mm. The algorithm accurately assessed the defect size, yielding a result of 24.3 mm. Shearography has several drawbacks, including challenges in inspecting deep or small defects and accurately characterizing the type of defect and its impact on material integrity [30].

Ultrasound testing (UT) is often used for various defect identifications. Specifically, air-coupled ultrasound has found an increasing interest in the inspection of honeycomb sandwich structures due to the ability to inspect a part without the need for a water couplant. Zhang et al. [31] utilized this method to detect defects in a honeycomb panel interface. They could detect 30 mm and 35 mm disbonds in the interface and measure them via the K-mean clustering method. Hsu [32] utilized air-coupled ultrasound to detect various defects at the interface within a sandwich structure but did not seek to quantify them geometrically. Zhou et al. [33] investigated CFRP honeycomb sandwich panels with an aluminum core via air-coupled ultrasound. Due to the large acoustic impedance between the air–solid interface, they were presented with a poor signal-to-noise ratio (SNR). Therefore, they introduced a hybrid method using a combination of wavelength filtering and phase-coded compression to improve the SNR.

Another interesting NDT method is the immersion-type UT scanning system. Several studies have utilized this method; however, none have specifically examined honeycomb structure panels with the objective of quantifying the defect dimensions. Blanford and Jack [34] used high-frequency ultrasonic to quantify the three-dimensional damage zone in a carbon fiber laminated composite from low-velocity impacts using immersion tank inspection. Blackman et al. introduced a novel pulse–echo ultrasound technique for sizing foreign objects in carbon fiber laminates [35], and recently, a study by Pisharody et al. [36] used immersion tank inspections to perform ultrasonic scanning to assess the damage zone around an adhesive joint. 

In light of the critical role that honeycomb sandwich panels play in diverse industries and the challenges posed by defects in their structural integrity, this study aims to investigate the effectiveness of single-side access, point-focused, ultrasonic NDT methods for defect detection and characterization. Specifically, the study is performed using spherically focused acoustic transducers in an immersion tank, as well as in a novel portable system [37] that provides the accuracy of an immersion tank without the need for submerging a component in water. This research seeks to enhance the understanding of defect identification in honeycomb panels and contribute to the development of reliable inspection techniques. This paper begins by outlining the manufacturing method employed for this study, followed by a concise overview of the inspection methods used for defect detection in honeycomb sandwich panels. Subsequently, the scanning setup is elucidated to provide insight into the experimental process. The next phase involves the analysis of the data captured in the preceding steps. Finally, the paper concludes by summarizing the key findings and implications drawn from the research. In this study, sizes of defects range from 5 to 40 mm and include missing adhesive, crushed core, removed core, embedded Kapton film, and embedded Polytetrafluoroethylene (PTFE, commonly termed Teflon) film. These defects are particularly challenging to detect, yet they were successfully identified and, more importantly, quantified, with an average error of only 0.6 mm over all coupons investigated, thus opening new possibilities in design and manufacturing, allowing for tighter tolerance manufacturing and better control over the required factor of safety margins.

## 2. Materials and Methods

### 2.1. Manufacturing Method

**Figure 1:**
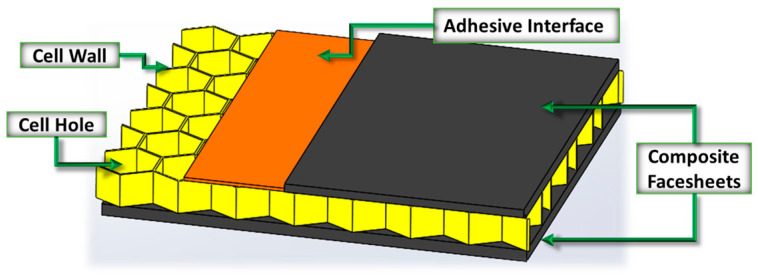
Representative schematic of honeycomb core material with carbon fiber facesheets.

**Figure 2:**
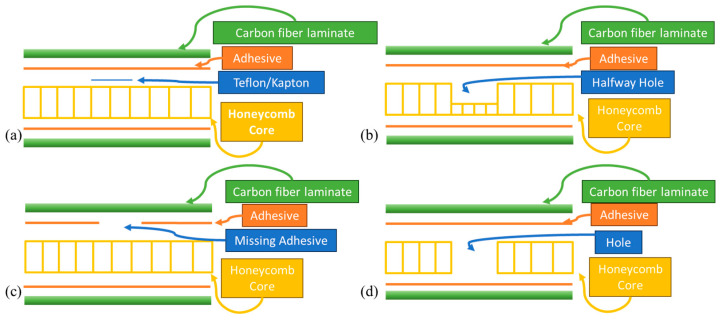
Representative schematic of defects placed within the manufactured coupon, (**a**) foreign object debris, (**b**) crushed honeycomb, (**c**) missing adhesive, and (**d**) removed honeycomb core.

Honeycomb sandwich panels (HSP) consist of three components: two facesheets and one honeycomb core, as illustrated in Figure 1. Facesheets can be constructed from various materials such as CFRP, fiberglass reinforced polymers (FGRP), or aluminum, while the core may be composed of Nomex or aluminum. In the present study, the fabrication of HSP is made of a CFRP facesheet composed of unidirectional 250 F pre-preg composed of Toray T700 fibers supplied by Rockwest Composites (West Jordan, UT, USA) and a Nomex core that is 25 mm in thickness and a nominal cell wall thickness between 0.2 mm and 0.4 mm. The process involves two main steps: preparing two facesheets within a Carver Laboratory programmable hot press (Wabash, IN, USA) following the manufacturer’s recommended cure cycle. Each facesheet is adhered to the honeycomb core using a 150 g pre-preg adhesive film from Easy Composites (Stoke-on-Trent, UK), again using the hot press to provide a controlled temperature and holding pressure. Various materials and methods are employed intentionally to simulate delamination between the facesheet and core. The unidirectional facesheets are manufactured with a layup of ${\left[0,45,-45,90\right]}_{s}$, where the subscript s stands for symmetric, yielding a total of 8 laminae for each facesheet with a nominal manufactured thickness of 1.2 mm. Prior to adhering the facesheets to the core, intentional defects are added, specifically those shown in Figure 2, including missing adhesive, a foreign object between the adhesive and the core, a partially damaged core, and a section of removed core. The partially damaged core is crushed with a flat bottom cylinder to one-half of the overall part thickness. These defects are selected due to their prevalence in the manufacturing process, as well as to highlight the ability to differentiate between defect types with the presented inspection method. The summary of the various defects for the 15 coupons, along with their dimensions, is provided in Table 1. The missing adhesive is created by physically removing a circular section from the adhesive film, simulating either an improper transfer of adhesive to the layup from the adhesive backing material or an air bubble preventing an adhesive from adhering to the facesheet. The foreign objects are made from either PTFE or Kapton; both materials are found within a standard manufacturing process [35]. The damaged core material is made by crushing the top surface of the core with a circular weight, simulating damage caused by improper handling during manufacturing. The final defect, the removed section of the core, simulates a purposeful removal of material as might be called out during manufacturing. The final planar dimensions of the coupon are nominally 75 mm × 75 mm, but the actual inspection area is typically much smaller, as will be noted in the following sections. Each of the defects was measured during manufacturing and prior to final assembly using a Keyence VR-3200 (Itasca, IL, USA) optical microscope to confirm proper manufacturing, record the defect for quality assurance, and capture any subtle dimension variations from the designed defect size. The dimensions for the various defects are provided in Table 1.

**Figure 3:**
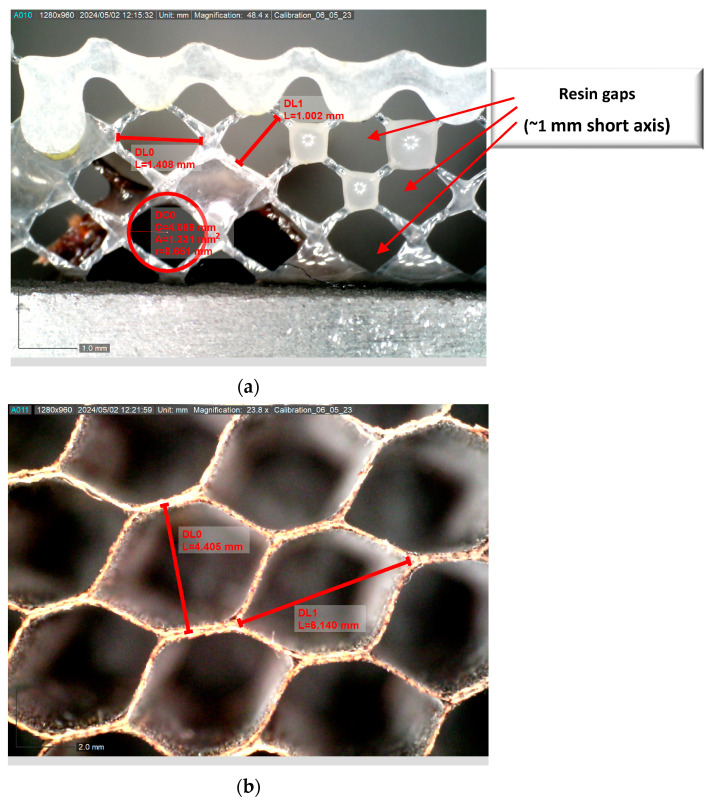
Microscope image of the adhesive film showing the associated punched holes allowing air pathways between layers (**a**) focusing on the resin adhesive sheet and (**b**) focusing on the honeycomb cell walls.

A unique feature that will be observed within this study inadvertently comes from the choice of the adhesive film. The adhesive film utilized incorporates small holes within the film, allowing air to pass between layers during manufacturing and aiding in the void removal process. These small holes appear in a regular pattern with an approximate spacing of 1 mm, as shown in Figure 3a. The microscope image of Figure 3a is after curing; thus, the resin will tend to wick along the various strands and between strands during the elevated temperature portion of fabrication. These small gaps do not impact the performance of the adhesive in the current application but are noteworthy, as they can be seen in the inspection results, as will be shown in Section 3. Of note is that these features that are 1 mm in span are smaller than the cells of the honeycomb, as shown in Figure 3b, which are typically 4.5 mm along the minor axis and 6 mm along the major axis. By observing these smaller features, one can have a sense of the effectiveness of the present system in identifying defects between the honeycomb cells and the facesheet. This high-resolution topic is briefly expanded upon in Section 3.3, where a comparison is made to the results from a roll-on adhesive, but it is not a prime focus of the current study.

### 2.2. Inspection Methodology

#### 2.2.1. Ultrasound Methodology

Ultrasonic testing (UT) is a nondestructive testing method commonly used for defect detection, part qualification, and evaluation of materials and structures. It relies on the propagation of high-frequency sound waves through the material being tested. These sound waves are introduced into the material using a piezoelectric transducer. All testing is performed using the same broadband spherically focused 15 MHz peak frequency transducer made by Evident (formally Olympus, headquartered in Waltham, MA, USA) with a focal length in water of 38.1 mm and a manufacturer-provided—6 dB loss beam diameter of focus, also termed the spot size, in water of 0.3 mm, which will nominally be 0.3 mm on the interface between the facesheet and the adhesive. Digitization is performed using a Focus PX, also from Evident, and spatial control is performed using Velmex (Bloomfield, NY, USA) linear translation stages. The digitizer fires a square wave pulse at 33 ns with a peak voltage of 190 V (the maximum voltage allowed by the hardware), and sampling occurs at 100 MHz. All inspections are performed with access to only a single side in what is termed pulse–echo mode. Unlike contact transducers, the spherically focused transducer is offset from the part surface. In the present study, instead of focusing on the front surface of the part, the focus is on the back surface of the facesheet using a surface offset $t_{focus}$ of

(1) $$t_{focus}=2\;w_{L}/c_{w}$$

where $c_{w}$ is the speed of sound of water and $w_{L}$ is the length the signal travels in the water, given as

(2) $$w_{L}=f_{L}-m_{T}\frac{c_{m}}{c_{w}}$$

In the above, $f_{L}$ is the focal length of the transducer, 38.1 mm in the present study, $c_{m}$ is the speed of sound of the CFRP of 2800 m/s, and $m_{T}$ is the material thickness of 1.2 mm.

**Figure 4:**
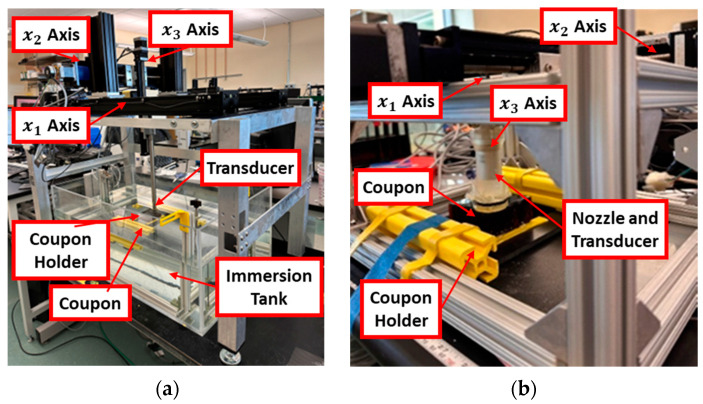
Custom immersion testing systems utilized in the present research. (**a**) Immersion scanning inspection system. (**b**) Portable (out-of-tank) inspection system.

As this study uses a spherically focused transducer, an acoustic medium, specifically water, is required between the transducer and the coupon being inspected. It is commonly assumed that the highest resolution ultrasound can be obtained using an immersion tank setup, such as that shown in Figure 4a. Unfortunately, these systems are constraining due to the need to submerge a coupon, unlike the air-coupled systems. We present a compromise in the present study with the portable inspection system presented in [37] and shown in Figure 4b. This portable system allows inspections to be performed in the field or the manufacturing environment without the need for submerging a coupon.

#### 2.2.2. Scanning System Set Up—Immersion Tank Inspection

**Figure 5:**
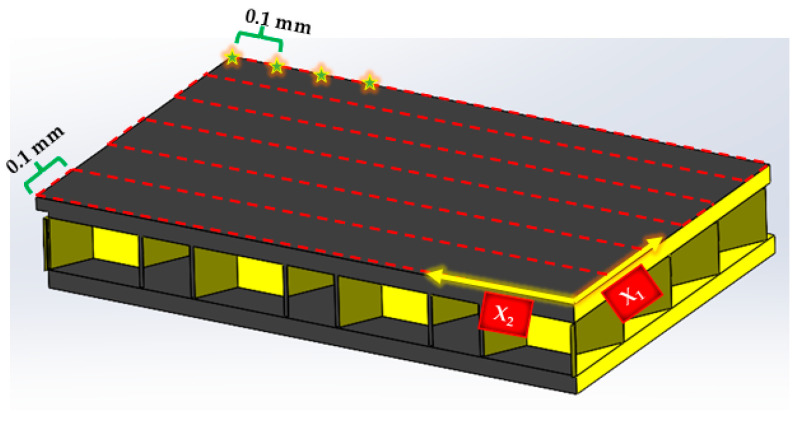
Raster pattern and coordinate axis utilized in scanning, with the red dashed lines indicating each of the individual scans separated by an indexing of 0.1 mm and the stars representing 4 of the over 600 points separated by 0.1 mm in each line scan.

Subsequently, the coupon is positioned within the immersion tank system shown in Figure 4a, where the entire surface area of each coupon undergoes scanning in a raster pattern, as depicted in Figure 5. The immersion system requires the complete submersion of the coupon, and a bagging material is placed around the edges of the honeycomb and sealed with gum tape to prevent water incursion of the coupon. The focal point of the 38.1 mm focal length transducer is at the back of the top facesheet and accounts for the impedance mismatch between the water and the composite when focusing. The raster pattern is executed in increments of 0.1 mm in both the $x_{1}$ and $x_{2}$ directions. The captured data are stored for subsequent analysis, facilitated by a custom in-house MATLAB script, discussed in Section 2.3, allowing for the identification, characterization, and interpretation of the various defects listed in Table 1 within the HSP interface.

#### 2.2.3. Scanning System Set Up—Portable System (Out-of-Tank) Inspection

One limitation of UT immersion tank systems is their inability to be easily deployed. Therefore, this study aimed to demonstrate the effectiveness of the method employed here using a novel portable UT system, which is discussed in detail in [37]. Figure 4b illustrates the setup, wherein a fixture has been specifically designed to accommodate the transducer within a captured water column [37]. In the present study, this is the same transducer used for the immersion tank inspections. This captured water column is then placed in contact with the surface of the coupon with an acoustic membrane between the water column and the coupon surface. A thin film of gel is then used to allow for acoustic coupling between the portable housing system and the coupon being inspected. Unlike traditional contact inspection methods where the resolution is like the transducer surface area, the present instance uses a spherically focused transducer, allowing for point inspections, thus maintaining the resolution advantage of the immersion systems with the portability and infrastructure advantages of hand-held contact elements.

### 2.3. Data Reduction Methodology

**Figure 6:**
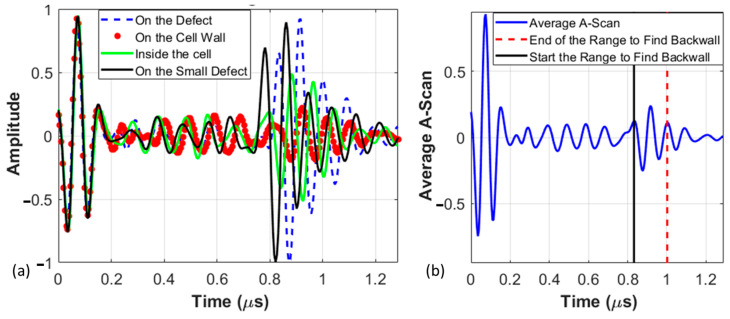
A-scans from Coupon 2, (**a**) at select locations and (**b**) the averaged A-scan.

In this investigation, data are captured by the Focus PX system purchased from Evident. The Focus PX system allows for both single-element inspections and phased array inspections with a 100 MHz digitizer with full integration capabilities for standard immersion testing systems. Data are collected and stored in the form of individual A-scans depicting the amplitude (strength) of the ultrasonic signal against the time taken for the ultrasonic pulse to traverse through the material, such as that shown in Figure 6 for Coupon 1, a sample with missing adhesive. However, in intricate structures such as the current honeycomb sandwich panels, A-scans alone are difficult to interpret and analyze. For example, Figure 6a displays A-scans taken at four different locations. The first is the blue dashed line, a typical A-scan over the defect, in this case, missing adhesive. The second is an A-scan taken directly over the cell wall but with adhesive, shown by the red dotted line. The third is a typical A-scan between the honeycomb walls but with proper bonding to the adhesive, indicated by the green line. The final A-scan, shown by a black line, is between the honeycomb walls but also in the gap between the perforations of the adhesive presented in Figure 2a. While the front wall of the top facesheet is visible in all A-scans at 0 μs, extracting useful information from a single A-scan proves challenging. Notably, the signal over the missing adhesive region, as well as in the adhesive region but between the perforations of the adhesive, the signal is similarly strong at the back wall of the facesheet near 0.8~1 μs. Conversely, the signal over the honeycomb walls with proper adhesive is weak at the interface. This signal is only slightly different than the signal over the region with adhesive but without the honeycomb cell wall. Consequently, discerning the exact flaw location depth or identifying the back wall of the top facesheet and flaw detection becomes difficult. To aid in the analysis, an average A-scan, termed $S\left(t\right)$, over the entire scanned region of the coupon was conducted for each of the individual coupons. This is defined as

(3) $$S\left(t\right)=\frac{1}{N_{1}N_{2}}\sum_{k=1}^{N_{1}}\sum_{l=1}^{N_{2}}S\left(x_{1,k},x_{2,l},t\right)$$

Here, $S\left(x_{1,k},x_{2,l},t\right)$ represents the ultrasonic signal intensity measured by the transducer at the *k*th location along $x_{1}$ where $k\in \left\{1,\;2,\;\ldots ,N_{1}\right\}$, and the lth location along $x_{2}$ where $l\in \left\{1,\;2,\;\ldots ,N_{2}\right\}$ at a specific time t. A typical result of the average A-scan is shown in Figure 6b. The black and re-dashed lines indicate the range from which to select for later processing to gate the back wall signal.

After capturing the waveforms over the scan region, the time axis on each scan is shifted such that the front wall echo occurs at the same time over all spatial positions in $\left(x_{1},x_{2}\right)$ such that the first echo occurs at the effective time of 0 μs, as shown in Figure 6. For each spatial location $\left(x_{1,k},x_{2,l}\right)$, the scan intensity is examined to identify the initial occurrence of a value exceeding a predefined detection threshold. Subsequently, the first peak after this time value is identified as the front wall echo for that location and a third-order polynomial in $\left(x_{1},x_{2}\right)$ is constructed to shift the time t by $t_{0}\left(x_{1},x_{2}\right)$ as $\hat{t}=t-t_{0}\left(x_{1,k},x_{2,l}\right)$. Thus, the ultrasonic signal intensity is investigated as $S\left(x_{1,k},x_{2,l},\hat{t}\left(x_{1,k},x_{2,l}\right)\right)$, where $\hat{t}\left(x_{1,k},x_{2,l}\right)=0$ corresponds to the front coupon surface.

**Figure 7:**
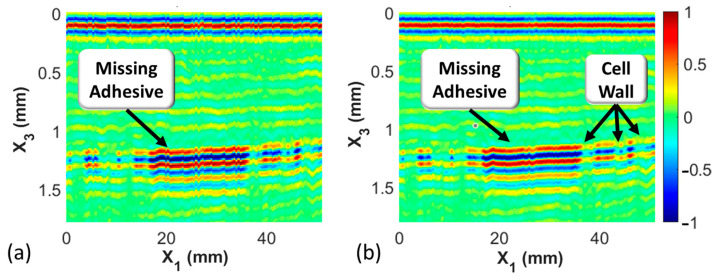
B-scans from Coupon 2, (**a**) without upsampling and (**b**) an upsampling factor of L=2 (color bar represents the normalized signal intensity).

To enhance the quality and accuracy of the data, a frequency upsampling technique is employed using the inverse Fourier transform. The upsampled frequency spectrum can be expressed as

(4) $$X_{u}\left(f\right)=\left\{\begin{array}{cc} L\cdot X\left(\frac{f}{L}\right) & for\;0\leq f<\frac{1}{L}\\ 0 & otherwise \end{array}\right.$$

where the upsampled frequency spectrum $X_{u}\left(f\right)$ is obtained by zero-padding the original spectrum and scaling it accordingly. Here, the capital letter $X\left(f\right)=F\left\{x\left(t\left(f\right)\right)\right\}$ denotes the Fourier transform of the original signal $x\left(t\right)$, f represents frequency, and L is the upsampling factor. Subsequently, interpolation is performed in the frequency domain, followed by the application of inverse Fourier transform on the interpolated spectrum to yield the upsampled signal.

(5) $$x_{u}\left(t\right)=F^{-1}\left\{X_{interp}\left(f\left(t\right)\right)\right\}$$

where $X_{interp}\left(f\right)$ is the interpolated spectrum. The results of this are shown through the B-scans shown in Figure 7, where the color bar indicates the normalized signal intensity. The B-scan is a collection of A-scans along a single axis, in this case along the index or $x_{1}$ direction. In Figure 7a, the raw scan is shown, whereas the upsampled scan is shown in Figure 7b, where the upsampling factor L is 2.

**Figure 8:**
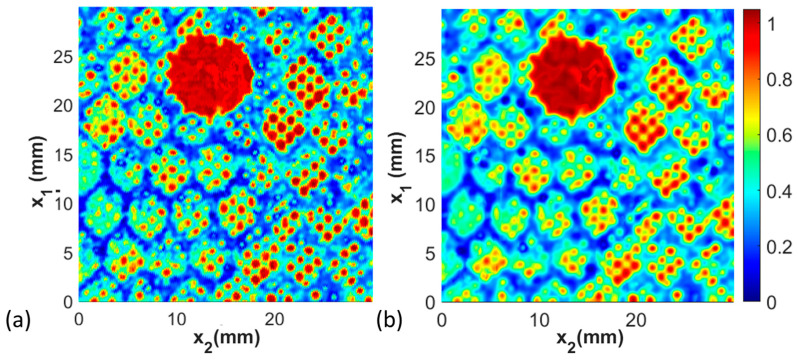
C-scans of Coupon 2, (**a**) prior to Gaussian filtering and (**b**) after Gaussian filtering.

The next step in this study involves the application of a Gaussian smoothing filter to smooth spatial variations. This method is employed to mitigate random noise present in the A-scan data [35] as

(6) $$\tilde{S}\left(x_{1,k},x_{2,l},\hat{t}\right)=\frac{1}{2\pi \sigma_{x_{1}}\sigma_{x_{2}}}\int_{-\infty }^{\infty }\int_{-\infty }^{\infty }e^{\frac{{-\left({\tilde{x}}_{1}-x_{1,k}\right)}^{2}}{2\sigma_{x_{1}}^{2}\;}}e^{-\;\frac{{\left({\tilde{x}}_{2}-x_{2,l}\right)}^{2}}{2\sigma_{x_{2}}^{2}}}S({\tilde{x}}_{1},{\tilde{x}}_{2},\hat{t}\left({\tilde{x}}_{1},{\tilde{x}}_{2}\right))\;d{\tilde{x}}_{1}d{\tilde{x}}_{2}$$

where the variables $\sigma_{x_{1}}$ and $\sigma_{x_{2}}$ tend to smooth out spatial information locally, where they are selected to be 3 times the step size in the current study. A larger value of σ leads to a wider Gaussian distribution, resulting in more extensive smoothing. Conversely, a smaller value yields a narrower Gaussian distribution, preserving more detail in the signal. Figure 8 illustrates the C-scan both before and after the Gaussian filtering. Notice that the filtering provides a crisper image and the edges of boundaries between features are more pronounced. To obtain a C-scan, the filtered acoustic signal, $\tilde{S}\left(x_{1,k},x_{2,l},\hat{t}\right)$ is converted to a 2-dimensional representation by taking the maximum value of the signal between a prescribed range, termed a gate Δt, as

(7) $$C\left(x_{1,k},x_{2,l}\right)=\underset{\hat{t}\in \left\{t_{1}\left(x_{1,k},x_{2,l}\right),t_{2}\left(x_{1,k},x_{2,l}\right)\right\}}{\max}\left\{\left|\tilde{S}\left(x_{1,k},x_{2,l},\hat{t}\right)\right|\right\}$$

where the value for $t_{1}$ is the onset of the back wall, shown in Figure 6b, and $t_{2}=t_{1}+\Delta t$, where Δt=0.05 μs. Different gate widths were investigated, but the narrow gate of 0.05 μs was found to yield an image with the highest signal contrast in the current study.

The C-scan provides a means to visualize information at a consistent depth, whereas the B-scan, as shown in Figure 7, provides a slice of information into the coupon. For example, the B-scan shown in Figure 7b can be used to detect the depth and estimate the size of a feature within a material. As can be seen in the B-scan, the interface between the facesheet and the adhesive for the honeycomb occurs around 1.2 mm into the coupon, and the width of the feature on the back wall, in this case missing adhesive, ranges from a value of $x_{1}\sim 18$ mm to $x_{1}\sim 38$ mm.

All analysis is implemented in the MATLAB (version 2022b, Natick, MA, USA) programming environment. This code facilitates precise measurement and analysis of the detected defects, allowing for the characterization and evaluation of their size within the scanned coupons.

## 3. Results

The following section provides the results from each of the studies. The results from Table 1 are presented for the immersion system, both as a function of the defect size and as a function of defect type. Next, a brief study on the subtleties between adhesive types is presented, as well as a brief study on the use of the portable inspection system. Finally, a summary of the results is presented in Section 4 to highlight the anticipated error in identifying the defect diameter, a value found to be typically less than 0.6 mm, with the inspection of Coupon 5 yielding the highest error of only 1.3 mm. The results of Section 4 also highlight that the portable system and the immersion system yield a similar error over the medium-sized, nominally 20 mm diameter, defect samples studied.

### 3.1. Defect Size Study

**Figure 9:**
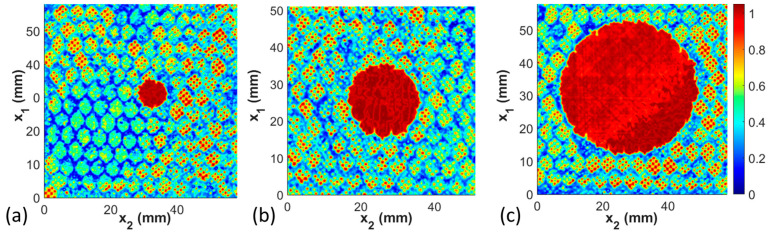
C-scans of Coupons 1–3, (**a**) 7.9 mm diameter defect, (**b**) 19.0 mm diameter defect, and (**c**) 40.8 mm diameter defect.

This first study presents the results for Coupons 1–3. These three coupons all have the same defect type, that of a missing adhesive, but have diameters of missing adhesive ranging from 7.9 mm to 40.8 mm. As can be seen in Figure 9, when properly gated, the defect is clearly identifiable. The diameter is manually measured three times by forming a best fit, in the visual sense, circle over the defect, and the average is then reported. For the three coupons in Figure 9 with the small (7.9 mm), medium (19.0 mm), and large (40.8 mm) diameter defects of the missing adhesive, the measured diameters using the ultrasound signal are, respectively, 8.3 mm, 19.3 mm, and 41.4 mm. This is only an error of, respectively, 0.3 mm, 0.3 mm, and 0.4 mm. Recall that the step size is 0.1 mm in both the $x_{1}$ and $x_{2}$ directions; thus, the error is on the order of 3 to 4 pixels, similar in value to the estimated beam spread of the focused signal. A study was not performed to identify if there is any difference in accuracy in the index direction $x_{1}$ or the scan direction $x_{2}$, as the accuracy of the present results is measurably better than in previous studies. It is worth noting in the figures that the missing adhesive appears as a high-intensity signal. This is due to the acoustic mismatch between the composite facesheet and the air located within the honeycomb core region. The cell walls can be clearly seen within each of the three scan data sets, as indicated by the hexagonal structure pattern throughout the image, except in the region of the missing adhesive.

### 3.2. Defect Type Study

**Figure 10:**
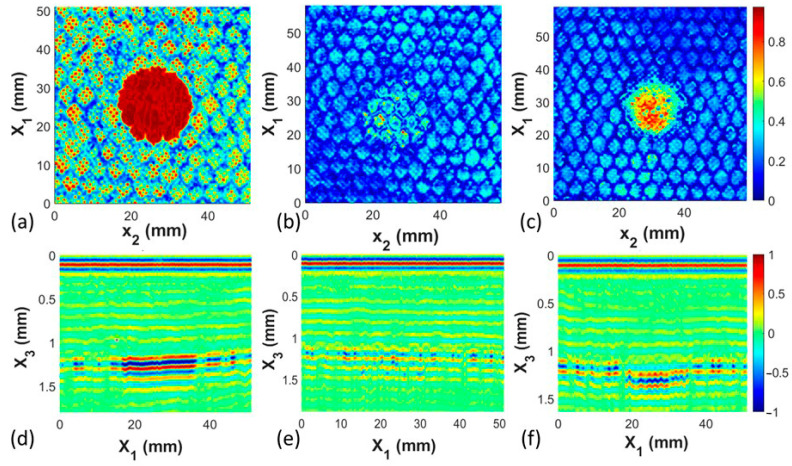
C-scans and B-scans for coupons with the nominal 20 mm diameter defect, (**a**) C-scan for Coupon 2, missing adhesive, (**b**) C-scan for Coupon 5, Kapton FOD, (**c**) C-scan for Coupon 8, missing honeycomb, (**d**) B-scan for Coupon 2, missing adhesive, (**e**) B-scan for Coupon 5, Kapton FOD, (**f**) B-scan for Coupon 8, missing honeycomb.

**Figure 11:**
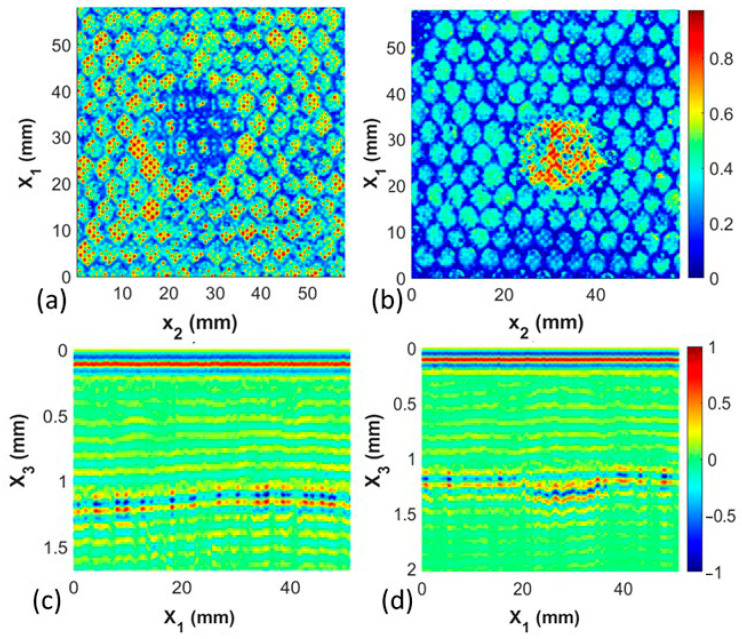
C-scans and B-scans for coupons with the nominal 20 mm diameter defect, (**a**) C-scan for Coupon 11, PTFE FOD, (**b**) C-scan for Coupon 14, crushed honeycomb, (**c**) B-scan for Coupon 11, PTFE FOD, (**d**) B-scan for Coupon 14, crushed honeycomb.

The second parameter of interest is the ability to differentiate between defect types. Five different defect types were studied, and the results from the medium-sized defects, which range from 19 to 20 mm in diameter, are presented in Figure 10 and Figure 11. The five types include two different types of foreign objects, Kapton and PTFE films, shown, respectively, in Figure 10b,e, and in Figure 11a,c. Observe that the circular defects/features are both visible in the gated C-scans on the back surface shown in Figure 10b and Figure 11a, but it is the B-scan at the back wall of the facesheet that can be used to differentiate the material type. Specifically, the Kapton film, shown in Figure 10e, has a soft acoustic reflection at the back wall located around 1.2 mm into the coupon as sound can pass through the interface between the Kapton and the surrounding material due to the light bonding of the resin with the film. Conversely, the acoustic reflection for the PTFE insert is much higher due to the lack of bonding between the PTFE and the surrounding polymer systems, as observed in Figure 11c. The missing adhesive coupons, shown in Figure 10a,d, are the clearest to see in the study, as the acoustic echo occurs earlier in time from the interface between the back of the facesheet and the air trapped in the honeycomb. The crushed honeycomb, Figure 10c,f, and the removed honeycomb, Figure 11b,d, are indistinguishable from each other. Although there are subtle differences in the C-scans in Figure 10c and Figure 11b, these are artifacts of the gating selected. Looking at the back wall of the B-scans in Figure 10f and Figure 11d, it is unclear if the differences are due to the different defect types or the resulting variability in manufacturing.

### 3.3. High-Resolution Capability and Variations in Adhesive Layer

**Figure 12:**
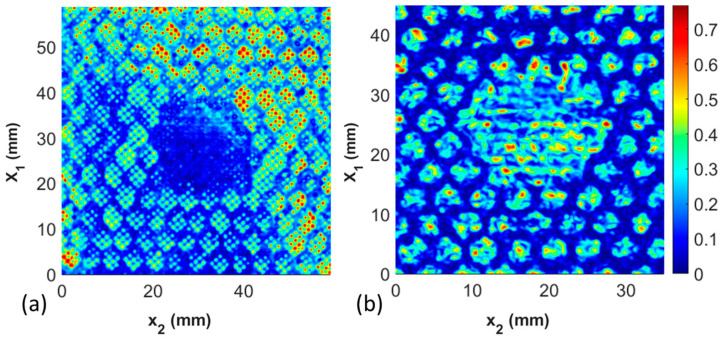
C-scan comparison between coupon with a nominally 20 mm diameter missing honeycomb defect made (**a**) with film adhesive with perforations and (**b**) with roll-on paste adhesive.

This next study focuses on the small features present in the C-scan waveform between the honeycomb cell walls seen in each of the previously shown C-scans. A sample with a 20 mm diameter missing honeycomb defect is shown in Figure 12a. Observe the small features on the length scale of 0.5 mm to 2 mm in the figure. These features have the same spacing as the perforations shown in Figure 3 of the film adhesive and are clearly visible in the final manufactured coupon. A second coupon with the same 20 mm diameter defect was fabricated, but a roll-on adhesive was used instead of the perforated film. The roll-on adhesive is significantly thicker than the film adhesive. During the elevated temperature step of curing, the honeycomb cells are pressed into the adhesive, causing a wicking of the resin on the honeycomb cell walls. This results in an apparent wall thickness significantly larger for the roll-on adhesive than for the film adhesive, as can be observed in Figure 12b. Of note is that the honeycomb pattern continues to be quite visible, but the crispness of the cell walls no longer exists as the reflections are dominated by the resin surrounding the honeycomb walls against the facesheet surface that wicked on the cell walls. Regardless, the 20 mm diameter defect is clearly visible in both adhesive systems, as noted in Figure 12.

### 3.4. Comparison between Portable Inspection System and Immersion Inspection System

**Figure 13:**
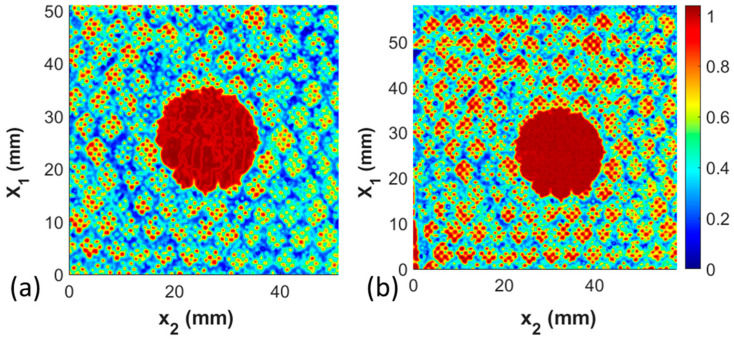
C-scan comparison between Coupon 2 with the inspection performed (**a**) in the immersion tank and (**b**) in the portable scanning system.

The final study is the comparison between the results taken from the high-resolution immersion system and the portable immersion system. The former requires the submersion of the coupon within water, whereas the portable system allows the coupon to be inspected in the field or the manufacturing environment without submersion in water. The results for Coupon 2, the missing film adhesive coupon with a 20 mm diameter defect, are shown in Figure 13a for the immersion inspection, and the portable inspection system results are shown in Figure 13b. The signal analysis methods of the captured data from both forms of data collection are identical. Observe that in both cases, the defect identified is graphically indistinguishable. Of note is that the smaller features, such as the cell walls and the perforations in the film adhesive, remain observable and quantifiable, but the resulting data set for analysis is not as clear for the portable system. Thus, the portable system can be readily used for the quantification of the primary defects sought after in the present study, but the smaller (less than 1 mm feature sizes) may be more difficult to quantify.

## 4. Discussion

The results shown in the previous section indicate the ability to capture defects that previously were considered undetectable using conventional ultrasound. Previous studies using Shearography were able to identify the existence of certain defects, specifically the largest defects investigated in the present study. Conversely, the present method allows for the detection of smaller defects and extends earlier works to allow for the quantification of the defect shape and size, as well as an empirical method to differentiate between defect types. In addition, many of the current studies focus on the probability of detection, or POD. In the present study, the medium-sized defects are often on the fringe of what is considered detectable; thus, one would expect a POD around the range of 50%. Using our presented method, we have a 100% POD over all samples studied, even the smallest defects which are considered undetectable. More importantly, we can dimensionalize the defect, something that is beyond the concept of POD but can be provided as a design tool for a structural engineer. The results for the 15 coupons from Table 1 are provided in Table 2, where the error is defined as the difference between the diameter measured using microscopy of the defect and the diameter extracted from the waveform data using the methods presented in Section 2. The error is defined as

(8) $$Error\equiv \left|d_{microscope}-d_{UT\;Inspection}\right|$$

The diameter reported in Table 2 is taken from the average of three tests of the same coupon to avoid any uncertainty due to human observations. A similar table is presented in Table 3, comparing the results for defect quantification from the portable system and from the immersion system. It was found that the average standard deviation by recharacterizing the captured data set for the immersion and portable system was 0.4 mm and 0.5 mm, respectively. The average error between the measured diameter between microscopy and the ultrasound inspection is 0.6 mm across all coupons for the immersion system, and it is 1.1 mm for the portable system. This is not significantly greater than the repeatability of the measurement itself of 0.4 mm. Thus, an improvement in resolution would be best obtained by refining the analysis method step that bridges the filtered and processed C-scan from Figure 8 to the quantification of the defect perimeter.

When looking at the defect quantification from the portable system and the immersion system, shown in Table 3, there is little difference between the results of the two systems. The portable UT system tended to yield slightly larger or smaller diameters across various defect types. For instance, it yielded the largest diameter measurements for missing adhesive defects. However, in PTFE, film defects showed considerable variation in diameter measurements between microscopy and portable UT. Additionally, the immersion system is more accurate than the portable system in various types of defects except Kapton film. In Kapton film, the portable system showed better results.

Of note in Table 2, there is no significant difference in the accuracy as a function of the size, where the standard deviation of error by size ranges from 0.5 mm to 0.6 mm. Conversely, there is an improvement in the accuracy for the missing adhesive coupons relative to the remaining coupons, with the missing adhesive having a standard deviation of error of 0.3 mm, whereas the standard deviation of the error for the coupons with missing honeycomb yields a value of 0.4 mm, and the remaining defect types yield a standard deviation of the error of 0.7 mm.

## 5. Conclusions

The presented study demonstrates the effectiveness of ultrasonic testing (UT) in differentiating between different defect types studied, including missing adhesive, embedded films, and core holes for honeycomb core structures. What we were unable to do was to differentiate between missing honeycomb and crushed honeycomb, but those two defect types are of a similar nature in that the honeycomb is not present under the surface of the CFRP facesheet. There is a high agreement between the characterized defect size measurements between the presented UT results and the microscopy images, with a typical difference between the methods of 0.6 mm. Of note, the ultrasonic immersion tank and portable systems exhibited the same inspection conclusions and both systems could identify all purposeful defects. Of interest was the unintentional identification of the film perforations that were on the order of 1 mm, a feature considerably smaller than the features of primary interest in the presented study. This is notable as it highlights the resolution capability of the presented methodology.

Overall, the findings underscore the significance of ultrasonic NDT methods in defect detection and characterization within honeycomb sandwich panels, with implications for ensuring the safety, reliability, and performance of composite structures across various industries. Moving forward, continued research and development in nondestructive testing techniques will further advance the understanding and capabilities in assessing the structural integrity of composite materials.

## Figures and Tables

**Table 1:** Test matrix of defect types and their associated planar dimensions.

Coupon Identifier	Defect Type	Diameter (mm) from Microscopy
1	Missing Adhesive	7.9
2	Missing Adhesive	19.0
3	Missing Adhesive	40.8
4	Kapton Film	4.7
5	Kapton Film	20.0
6	Kapton Film	40.3
7	Hole	5.7
8	Hole	18.8
9	Hole	34.8
10	PTFE Film	5.0
11	PTFE Film	20.0
12	PTFE Film	40.4
13	Crushed Hole	6.4
14	Crushed Hole	19.3
15	Crushed Hole	35.5

**Table 2:** Measurement error from ultrasonic characterization for each coupon.

Coupon	Defect Type	Diameter (mm) Microscopy	Diameter (mm) UT Results	Error (mm)
1	Missing Adhesive	7.9	8.3	0.4
2	Missing Adhesive	19.0	19.2	0.2
3	Missing Adhesive	40.8	41.1	0.3
4	Kapton Film	4.7	5.1	0.4
5	Kapton Film	20.0	21.3	1.3
6	Kapton Film	40.3	40.8	0.5
7	Hole	5.7	6.3	0.6
8	Hole	18.8	18.4	0.4
9	Hole	34.8	35.0	0.2
10	PTFE Film	5.0	4.2	0.8
11	PTFE Film	20.0	20.5	0.5
12	PTFE Film	40.4	41.3	0.9
13	Crushed Hole	6.4	7.2	0.8
14	Crushed Hole	19.3	19.1	0.2
15	Crushed Hole	35.5	34.3	1.2

**Table 3:** Inspection results for coupons with a medium (nominally 20 mm) defect inspected with the portable system and the conventional immersion system.

Coupon	Defect Type	Diameter (mm) Microscopy	Diameter (mm) Immersion UT Results	Diameter (mm) Portable UT Results
2	Missing Adhesive	19.0	19.2	20.3
5	Kapton Film	20.0	21.3	20.34
8	Hole	18.8	18.4	17.07
11	PTFE Film	20.0	20.5	18.7
14	Crushed Hole	19.3	19.1	18.58

## Data Availability

The original contributions presented in the study are included in the article, further inquiries can be directed to the corresponding author.
